# A Multidisciplinary Telerehabilitation Approach for Supporting Social Interaction in Autism Spectrum Disorder Families: An Italian Digital Platform in Response to COVID-19

**DOI:** 10.3390/brainsci11111404

**Published:** 2021-10-25

**Authors:** Ersilia Vallefuoco, Giulia Purpura, Giovanna Gison, Andrea Bonifacio, Luca Tagliabue, Fiorenza Broggi, Goffredo Scuccimarra, Alessandro Pepino, Renata Nacinovich

**Affiliations:** 1 SInAPSi Centre, University of Naples Federico II, 80126 Naples, Italy; 2 School of Medicine and Surgery, University of Milano Bicocca, 20900 Monza, Italy; giulia.purpura@unimib.it (G.P.); fiorenza.broggi@unimib.it (F.B.); renata.nacinovich@unimib.it (R.N.); 3 Department of Mental, Physical Health and Preventive Medicine, University of Campania Luigi Vanvitelli, 80138 Naples, Italy; giovanna.gison@unicampania.it (G.G.); andrea.bonifacio@unicampania.it (A.B.); 4 Child and Adolescent Health Department, S. Gerardo Hospital, ASST of Monza, 20900 Monza, Italy; luca.tagliabue@unimib.it; 5 Fondazione Istituto Antoniano, 80056 Naples, Italy; goffredo.scuccimarra@istitutoantoniano.it; 6 Department of Electrical Engineering and Information Technology, University of Naples Federico II, 80125 Naples, Italy; pepino@unina.it

**Keywords:** autism spectrum disorder, telemedicine, digital platform, individual-centered approach

## Abstract

Due to its complexity and high variability in symptomology, autism spectrum disorder (ASD) requires a coordinated and multidisciplinary intervention to better support the different programs over time and to promote social interactions in all contexts of life. Telemedicine can offer a valuable contribution in this regard, providing low-cost and portable applications. In this paper, we presented an Italian project, *SUPER*, which aimed to foster collaboration and information sharing between ASD families, health services, and schools. *SUPER* provided a digital platform with several tools that were useful both to enhance general and specific ASD knowledge and to promote personalized programs for children with ASD. We conducted a preliminary user test for the platform with 30 participants (18 therapists and 12 parents of children with ASD) using the system usability scale (SUS). The total mean SUS score (89.2) showed that *SUPER* is an excellent, usable system. Moreover, we extracted the usability and learnability mean components from the SUS scores, which were 96.1 and 61.7, respectively. Our preliminary results indicate that *SUPER* is a very user-friendly application and its innovative telemedicine approach could be ahelpful communication and collaboration tool among the different contexts of care for children with ASD.

## 1. Introduction

Autism spectrum disorder (ASD) is an early-onset neurodevelopmental condition characterized by significant impairments in social interaction and communication, restricted interests, and repetitive and stereotyped behaviors [1]. Although the complexity of this condition involves several abilities and learning impairments, a social interaction deficit is considered the core symptom for its impact on adaptation to surrounding environments across the lifespan of an individual and for its cascading effects on all other neurodevelopmental domains [2,3]. It is now well established from a variety of studies that parents or caregivers of ASD children have an increasing risk of high stress levels and psychosocial problems [4,5], and that the severity of ASD symptoms is one of the most important factors affecting the caregiver burden in this population [6].

For these reasons, the importance of early, multidimensional, and family-centered interventions was highlighted by several research groups [7,8], and the need for multidisciplinary teams to ensure a complete and prolonged care over time was widely recognized [9,10,11]. As suggested by [8,10,11,12,13,14], a multidisciplinary approach should involve all the people that are directly involved in the individual’s life, including teachers and support workers. On the one hand, schools can be a great opportunity for these children, being a social context where relationships and continuous exchanges thrive, while on the other hand, dealing with ASD-related issues, especially those involving social interaction skills, is not so easy. Moreover, activity limitations should not preclude children or adults with neurodevelopmental conditions from healthy living and social participation, since life experiences can enhance their opportunities to learn and develop skills for current and future participation in desired social roles [15]. Therefore, it is crucial to support a joint collaboration and coordination among all of the parties involved to define a life project for the individual with ASD.

However, in clinical and educational practice, this multidisciplinary cooperation is not always achieved, and an asynchrony between several inhomogeneous and inconsistent interventions is very frequent [16]. In addition, this approach was further complicated in January 2020 when the World Health Organization declared the outbreak of a coronavirus disease from 2019 (COVID-19), a public health emergency of international concern [17]. From that moment on, groups of researchers and clinicians from all parts of the world have tried to continue the work of early diagnosis and intervention of ASD to guarantee the rights to health for these children and their families despite the global emergency [18], even though the lockdown period was very hard on these families, resulting in an increase in the stress and burden levels [19,20]. The two most critical points of this period were arguably both the decrease in opportunities for social participation in normal life activities and the different and more difficult ways of communicating between family, health, and school services.

There is a growing body of literature that recognizes the role of telemedicine in ASD diagnosis and interventions, especially during the COVID-19 emergency [18,21,22,23,24,25]. For instance, Narzisi [18] proposed a telemedicine working model for the preliminary diagnosis and intervention of children with ASD, using synchronous and asynchronous transmission. Conti et al. [21] illustrated a detailed remote surveillance protocol to support ASD early diagnosis, whereas Wagner et al. [25] described a model of teleassessment for ASD in young children, providing promising preliminary results. However, although telemedicine has provided an important support to deal with ASD and other neurodevelopmental disorders families [26,27], this approach has often offered an exclusive relationship between clinicians and patients, with reduced involvement of other community services [28] to the disadvantage of multidisciplinary work. In addition, as suggested by [21,27,28], telehealth usage requires user-friendly, low-cost platforms that are accessible to all and safeguard data security in compliance with the data protection rules. Moreover, the majority of technological systems were not designed to support several interventions for people with ASD, but adapted to that purpose.

To respond both to the difficulties caused by the pandemic and pre-existing problems, we accelerated the development of a new Italian project: *SUPER*. The *SUPER* (*Sistema Unitario in una Piattaforma Riabilitativa ed Educativa*, that is, a joint system in a rehabilitation educational platform) project tries to support multidisciplinary work, focusing on the use of telemedicine to ensure synergy between ASD families, health services, and schools. In particular, *SUPER* relies on a digital platform providing different tools, both common use and designed ad hoc, to efficiently and easily share rehabilitative and educational objectives and strategies with low-level costs for families and services. It was designed following the ASD International Guidelines [8,10,11,12,13,14] and the recommendations of the International Classification of Functioning, Disability and Health for Children and Youth (ICF-CY) [29]. Moreover, *SUPER* was engineered to be easily accessible and usable, protecting the security and safety of data management. *SUPER* aims to: (i) facilitate the sharing of science-based information on ASD; (ii) promote an individualized approach to handle children (aged 3–10 years) that were diagnosed with ASD; (iii) create a common language between parents, teachers, and clinicians using the ICF-CY framework; (iv) promote the sharing of functioning profiles of individuals with ASD in different life contexts; and (v) support the identification and sharing of rehabilitative and educational purposes.

In the current paper, we provide an overview of the platform’s structure and report the results from a preliminary usability test that was performed using the system usability scale (SUS) [30], which will be helpful to evaluate a possible redesign of our system in order to validate its efficacy.

## 2. Materials and Methods

To design *SUPER*, we followed the main recommendations from the ASD International Guidelines [8,10,11,12,13,14]. We adopted an individual and family-centered approach, where professionals had to cooperate with individuals and their families in all aspects, from assessment to intervention strategies, to life project planning [8,11,13]. Continuous information sharing between the professionals and the families also had to be ensured [8]. Additionally, clinicians had to inform and educate the families on ASD (e.g., providing manuals, booklets, and informative materials), acting as coaches for the people directly involved in the individual’s life. In fact, it should be good practice to always identify a case manager for each individual with ASD [11].

In accordance with this approach, and considering the wide heterogeneity of ASD, the ASD International Guidelines [8,10,11,12,13,14] also recommend highly individualized programs based on both the needs of the individual and the best possible outcomes they can achieve. All of the actions should thus be both personalized on the characteristics of each individual with ASD and adaptive, as they should change over time depending on the progress, treatment responses, and the developmental phase. In addition, all of the actions should be consistent and shared in all of the contexts of life, including education, social, health, and care settings.

In compliance with the Australian guidelines [13], we adopted the ICF-CY [29] as a holistic framework to design specific tools that were useful to describe each individual’s functioning profile, monitor progress, and establish joint purposes. After all, the ICF-CY framework provides a common and universal language that can be shared by all of the services and systems where the individual is involved.

### 2.1. The SUPER Platform

*SUPER* is an Italian digital platform that can be used on both desktop and mobile devices. The current setup for the platform was designed to help handle children with ASD aged 3–10 years. It was targeted at families of children with ASD, teachers, therapists, clinicians, and other professional figures of interest. We created three different user profiles for the several involved figures, as reported in Table 1. Access to the different sections and actions that could be performed in the platform was personalized for each user profile. During the registration process, users had to indicate their role and define their username and password to log in to the platform.

**Figure 1:**
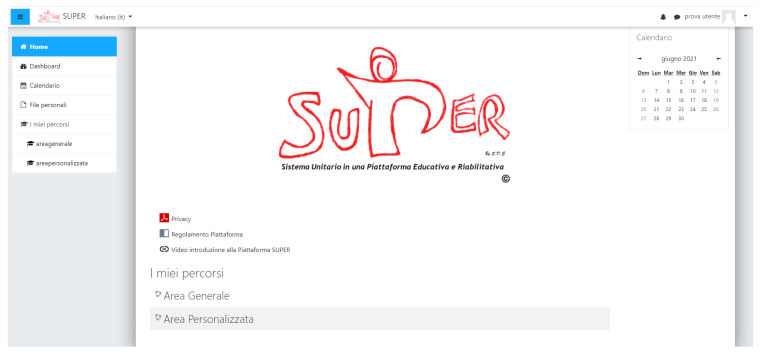
Screenshot of the *SUPER* homepage. The figure features the homepage of the *SUPER* platform, where users can access the General and Personalized Area.

As shown in Figure 1, the platform was organized into two main areas: General and Personalized. The General Area aimed to promote general scientific knowledge on ASD and to respond to the diverse educational-informative needs of the platform’s users. It included science-based information on ASD, delivering contents that were made by Italian experts specifically for the *SUPER* project. This material consisted of videos, slideshows, and other documents that could be viewed by all of the users, regardless of the user type. Moreover, content was gathered in three main sections:

- Understanding ASD: providing information on ASD and its clinical features (including etiopathogenesis, epidemiology, symptoms, diagnostic tools, interventions).
- Contexts: providing information on how ASD can affect different life contexts (including family, school, clinical settings, society).
- Research material: providing content on specific topics.

**Figure 2:**
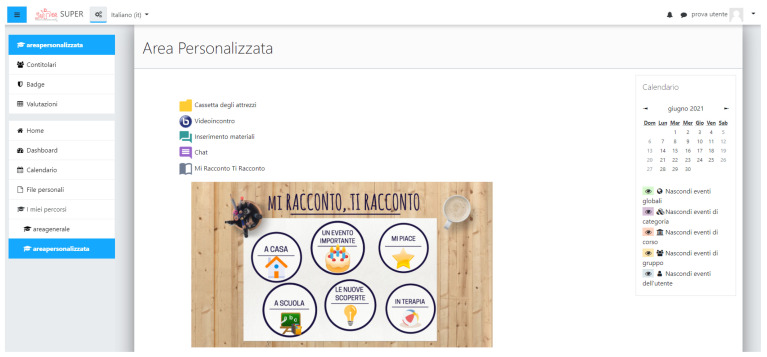
Screenshot of the initial page of the Personalized Area. The figure shows an overview of the Personalized Area, where useful communication and content sharing tools were included.

The Personalized Area (Figure 2) was a restricted space for groups of users following an individual with ASD. The purposes of this area were to encourage a personalized approach, to facilitate both group communication and material sharing, and to provide useful tools for evaluation and monitoring in different life contexts. We also established another user profile for this area, the case manager, who was assigned more privileges than other users. They were able to access all of the sections, further edit and customize the provided tools, and add new tools or activities based on the child’s needs and their programs.

This area was structured in a general section that was accessible to all of the user profiles and provided useful tools to communicate and share information and material among users. The users were able to chat for brief communications or video call for meetings and discussions, freely scheduling these activities in the platform’s calendar. One of the activities, *Material Upload* (*Inserimento Materiale*), allowed the users to upload and share material and information, especially short video clips, with other users. This activity was specifically created to support video-feedback strategies that can be useful both to improve the child’s communication and social skills and to evaluate their progress over time [31]. Another content sharing tool, *I talk about myself and I talk about you* (*Mi Racconto, Ti Racconto*), was included mainly to notify all of the users of important events and routine activities in the child’s life. It featured a photobook structure that was divided into five parts (at home, during therapy, at school, new discoveries, important events and hobbies). The users were able to upload photos with a short description for each unit, sharing a unique portrayal of the child and focusing on their strengths, rather than just their limitations. Furthermore, using images and photos can stimulate individuals with ASD to communicate and promote their autobiographical skills [10].

**Figure 3:**
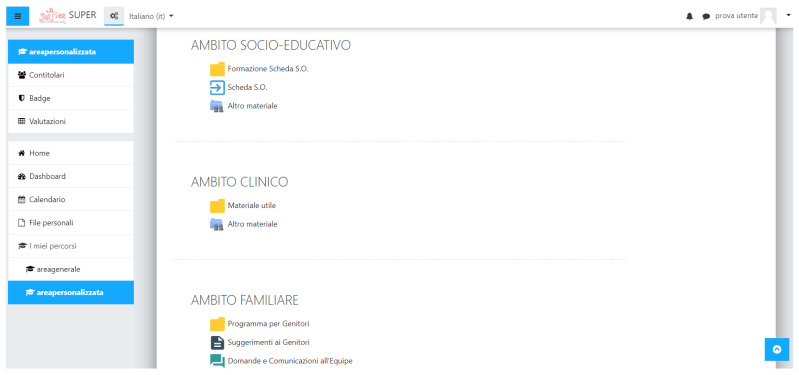
Screenshot of the different sections of the Personalized Area. The figure shows the three specific sections of the Personalized Area designed for each user profile.

In addition, the Personalized Area offered three sections (Figure 3) with different access levels depending on the user profile. The clinical profiles had unique access to all of the sections, including their own dedicated, customizable space where they could upload useful materials. The socio-educational section was mainly meant for users with a social and educational profile. For this section, we created a form gathering observations on individuals with ASD to improve and enhance cooperation between the clinicians and teachers. This *Observation Form* (OF, *Scheda di Osservazione* in Italian) was based on the ICF-CY framework and used specific ICF-CY codes from selected activities and participation domains. A performance qualifier was identified for each ICF-CY code, alongside the main items to consider to assign a value to the qualifier itself. In addition, items included a description correlating them to the typical developmental phases. The form had to be drafted and discussed by the clinicians and teachers during their meetings in order both to establish joint rehabilitation-educational goals that the child had to achieve and to supervise their progress. Finally, the last section of the Personalized Area was intended for users with a family profile. The aim of this section was to create a direct communication channel between the clinicians and families, while offering useful information and material. The parents could upload programs or therapies they were following, view a short summary of the OF, and ask confidential questions. On the other hand, the clinicians could provide advice to the parents using the *Suggestions to Parents* tool, which allowed them to enter written recommendations or instructions to follow at home. An overview of the different access levels for each user profile was reported in Table 2.

We provided instructions and videos on how to navigate the platform and its tools for a smoother user experience. The users could also contact and request technical assistance from the platform’s help desk. Moreover, all of the users had to go through a specific training on methodologies and tools before starting to use the platform.

*SUPER* was developed by customizing the open-source e-learning platform Moodle (modular object-oriented dynamic learning environment) [32]. Moodle allows educators and technicians to create a customized virtual learning environment, granting access only to enrolled users. It offers different synchronous and asynchronous communication features, assessment tools, and a wide variety of complementary plugins to support the users in performing their tasks. Since one of the pivotal elements of a good digital application is to be user friendly, Moodle does not require significant computer skills and provides a simple and intuitive user interface [33]. However, some of the options and tools were customized and adjusted to guarantee that all of the data gathered from *SUPER* were treated in accordance with the General Data Protection Regulation (GDPR EU, 2016/679) [34]. Additionally, the users had to sign our terms of use and a joint ownership agreement, as well as give their informed consent to the personal data treatment, in order to access the platform.

### 2.2. Usability Evaluation

We conducted a preliminary user test to understand *SUPER*’s perceived usability and gather feedback.

#### 2.2.1. Participants

For this study, 30 participants, specifically 18 therapists and 12 parents, were enrolled voluntarily from the Centro Medico Riabilitativo di Pompei, according to the following inclusion criteria: (i) Italian native speaker; (ii) capable of using a personal computer (PC) and mobile devices; (iii) for therapists: following children with ASD in a rehabilitation routine; for parents: having a child with ASD who was diagnosed according to the *Diagnostic and Statistical Manual of Mental Disorders*, Fifth Edition (DSM-5) criteria [1]. We did not require ethical approval for the usability test, but each participant was informed of the study and gave their consent to participate. The data were collected anonymously through a Google form. Moreover, we did not have the users register with a username and a password, but we gave them free access to the platform only for these usability test sessions.

#### 2.2.2. Procedure

Due to the COVID-19 restrictions, the study was conducted virtually, using the Zoom video platform. We carried out two different test sessions: one for therapists and one for parents; the participants of the same group performed the test simultaneously. We started off both sessions by explaining how they would work and introducing the *SUPER* platform. We provided only a short virtual tour of *SUPER*, describing the platform’s structure and tools for 10 min, with no instructions on how to use them. We used the same presentation and provided the same information for both of the test sessions. Subsequently, we asked the participants to access *SUPER* and use it for at least 30 min, but not more than 120 min, and to perform a set of tasks (reported in Table 3). During the test sessions, we did not interact with the participants in order to avoid influencing their user experience. After completing all of the required tasks, the participants had to fill out a Google form with the usability scale that was shared in the video call chat. Finally, we collected the general impressions and feedback from the participants.

#### 2.2.3. Outcome Measures

The platform’s usability was evaluated with the system usability scale (SUS) [30], Italian version [35], as reported in Table 4. The SUS is a standardized tool for measuring the perceived usability of an interactive system, such as websites, user interfaces, and web applications. It consists of 10 items that are scored on a Likert scale, from 1 (strongly disagree) to 5 (strongly agree). The total SUS score is calculated by subtracting 5 from the sum of all of the odd items and subtracting the sum of all of the even items from 25; these values are then summed and multiplied by 2.5 to obtain a final score ranging from 0 to 100. The SUS scores below 68 reveal usability issues with the system, while scores higher than 68 indicate acceptable levels of usability. More specifically, following the adjective grade ranking proposed by Bangor et al. [36], scores over 80 suggest an “excellent” usable system, scores between 68 and 80 can be considered “good”, scores between 51 and 68 indicate “fair” results, scores below 51 are “poor”, and scores below 36 reflect “unusable” systems. In addition, since our participants were experts in the application domains [37], we also performed a bidimensional analysis of the SUS scale, involving the usability and learnability subscales [38]. The usability subscale covers items 1, 2, 3, 5, 6, 7, 8, and 9 from the SUS scale, while items 4 and 10 are included in the learnability subscale. In order to calculate the usability and learnability scores from the SUS scores, the SUS summed score contributions must be multiplied by 3.125 for usability and by 12.5 for learnability, respectively [38].

#### 2.2.4. Statistical Analysis

A statistical analysis of the SUS scores was conducted using R (software version 4.1.1 for Windows), considering *p* < 0.05 as statistically significant. The mean scores and standard deviations were calculated for all of the participants and for the two user categories, therapists, and parents. Because of the small sample size, we combined a visual inspection and a normality test (the Shapiro test) to evaluate the score distribution for the SUS, usability, and learnability between the two groups and to establish whether to adopt a parametric or a non-parametric test.

## 3. Results

Overall, the participants did not have any particular issues during the test sessions, showing great interest in using *SUPER*. During the final discussions of the usability test, they highlighted how user friendly the platform is, especially for its clear structure and its simple user interface. As for the General Area, a particular interest was shown for the informative short video format, whereas the participants were quite intrigued by *I talk about myself and I talk about you* in the Personalized Area and viewed it as a potentially useful tool. The general SUS, usability, and learnability scores with their standard deviations are summarized in Table 5.

We detected an “excellent level” of system usability (M = 89.2, SD = 5.5) from the mean SUS score, according to Bangor et al.’s criteria [36]. Similarly, considering the two different user types, the mean scores for therapists and parents were 90.7 (SD = 5.1) and 86.9 (SD = 5.5), respectively (as shown in Table 5), indicating an “excellent” usable system. With reference to the SUS subscales, the total mean score for the usability subscale was 96.1 (SD = 4.6), with 97.1 (SD = 5.0) for the therapist group and 94.6 (SD = 3.8) for the parent group. The total mean score for the learnability subscale was 61.7 (SD = 12.5), with mean scores of 65.3 (SD = 9.1) and 56.2 (SD = 15.5) for therapists and parents, respectively.

**Figure 4:**
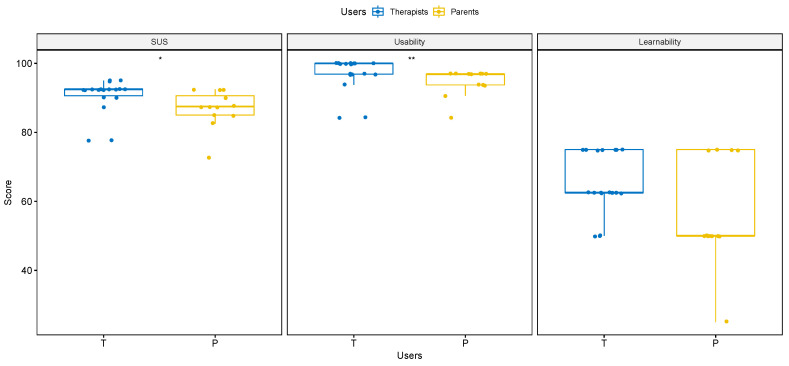
Comparison of mean scores between the two user groups. The figure reports the Mann–Whitney *U* test results, comparing the two user groups, therapists and parents, using the SUS, usability, and learnability scales. A significant statistical difference was detected only for the SUS (* = *p* < 0.05) and usability (** = *p* < 0.01).

To establish which test should be used to compare the two user groups, we visually analyzed the data and performed a Shapiro–Wilk test as a normality test. Both of the analyses (Shapiro-Wilk test: *p* < 0.05; therapist group: *p*${}_{\mathrm{SUS}}$ < 0.001, W = 0.65; *p*${}_{\mathrm{Usability}}$ < 0.001, W = 0.63; *p*${}_{\mathrm{Learnability}}$ < 0.01, W = 0.80; parent group: *p*${}_{\mathrm{SUS}}$ < 0.05, W = 0.83; *p*${}_{\mathrm{Usability}}$ < 0.001, W = 0.69; *p*${}_{\mathrm{Learnability}}$ < 0.01, W = 0.78) indicated that the data distribution was significantly different from the normal distribution. Therefore, a Mann–Whitney *U* test was conducted to compare the individual SUS scores for the therapist and parent groups. As shown in Figure 4, the Mann–Whitney *U* test reported a significant difference in the SUS (*p* < 0.05, U = 166, Â${}_{12}$ = 0.77) and usability (*p* < 0.01, U = 167, Â${}_{12}$ = 0.77) scores between the two user groups, with no significant difference (*p* > 0.05, U = 147.5, Â${}_{12}$ = 0.68) for the learnability score.

## 4. Discussion

Telemedicine was a good resource for the families and clinicians of children with ASD during the COVID-19 health emergency and its use might be encouraged and employed in several situations where communication and social interaction are hindered by external factors. In the present study, we illustrated a new Italian project, *SUPER*, whose main purpose was to improve the communication and collaboration among clinicians, teachers, and parents of children with ASD in order to support the different interventions and strategies in place. The project was designed on the main ASD International Guidelines, identifying and including strategies that should be useful in all interventions or treatments. Moreover, *SUPER* drew on the ICF-CY to describe the individual’s functioning and to provide a common standardized universal language to the different professionals involved. *SUPER* was supported by a digital platform providing not only general resources to understand and learn about ASD, but also specific tools to support the multidisciplinary teams following children with ASD.

In order to investigate the platform’s perceived usability, we carried out a preliminary usability test with 18 therapists and 12 parents of children with ASD, using the SUS as a usability scale. The usability test yielded positive results, with an “excellent” (>80) mean SUS score. We obtained similar results by analyzing the two different user groups. Alongside a unidimensional analysis of the SUS score, we conducted a bidimensional analysis, evaluating the usability and learnability subscales in accordance with Othman et al. [37]. With reference to the usability subscale, the mean score obtained (96.1) suggested there were no issues in the usability system; in fact, all of the participants, both therapists (97.1) and parents (94.6), described *SUPER* as very user friendly. There are two main possible explanations for this result: as reported by the participants at the end of the test, the structure was intuitive and clear, so the users were able to easily navigate the platform; *SUPER* likely met the users’ professional and personal needs, leading them to choose positive SUS items. The overall mean learnability score (61.7) suggested that this component was deemed only “fair” by users, so further studies are necessary to investigate this factor. However, in accordance with similar usability tests [37], this outcome is likely related to the participants not being given specific instructions on how to use the different tools.

A comparative analysis of the two user groups showed differences in the SUS and usability scores. Even though the therapists had access to more advanced tools and were assigned additional tasks, they perceived the platform as very user friendly. Therefore, the additional tasks and tools were not viewed as difficult and complex by clinicians. An analysis of the learnability subscale did not reveal a significant difference between the mean learnability scores, and all of the participants suggested reviewing this aspect.

Overall, our results suggest that *SUPER* is a highly usable system and indicate that users perceived it as an excellent platform for its purposes, but we also detected medium-low scores for learnability in both of the user groups. Therefore, despite the positive preliminary results, we plan to review certain elements of the platform to improve its usability, for example, providing additional video tutorials, material, and specific training to facilitate, in particular, learnability. Moreover, we want to include a brief description for each tool to help the users understand its functions.

During discussions with the participants, they highlighted the potentiality of the platform, especially for supporting communication and collaboration among the different professional figures involved in the rehabilitation of a child with ASD. In particular, the therapists praised the possibility to collect and share information, recommendations, and strategies in a unique and personalized virtual space. On the other hand, the parents commented on how the platform could be a useful tool to support them and their families beyond the rehabilitation context. These observations may support the hypothesis that *SUPER* can facilitate the interaction between the adults around children with ASD (parents, physicians, therapists, teachers, and other social service workers), reducing the barriers and aiding healthcare communications during care delivery. Better communication can help carry out the various interventions already in progress to reduce the social interaction deficit in these children, with possible neuropsychological improvements.

According to our participants, *SUPER* could be effective because it is simple to use, portable, and low cost. This aspect is very important in telerehabilitation systems; in fact, even before the pandemic emergency, researchers and clinicians had already started to suggest that telerehabilitation could be useful in the recovery of neurological and neuropsychological functions since it is cost effective and accessible to more patients [26,39,40]. On the other hand, as suggested by other studies [19,21,27], diagnosis and rehabilitation can be offered through computer or mobile devices not as a replacement for the common evaluation processes and therapies, but to integrate health services and make them more enjoyable by a virtuous coexistence of virtual and in-person assessments.

Our findings and considerations on this approach were also supported by Narzisi [18], who suggested that a good and well-organized telemedicine model could provide useful indications for psychoeducational interventions at home, given the complexity of ASD, where motor, sensory processing, and communication difficulties intersect from the earliest stages of life, making a multidimensional and multidisciplinary approach necessary [41,42,43,44]. For these reasons, *SUPER* was designed not to adhere to a specific intervention’s model but as a means of support for therapists and educators, to help them respond to the specific, individualized needs of children with ASD and their families with a development-based approach.

A limitation of this study is that the preliminary user test was conducted only on a small sample of therapists and parents. Therefore, our findings are limited and cannot be generalized to all of the user categories. While *SUPER* did not require technical skills to be used, we enrolled users that were already capable of using a PC and familiar with ASD in this preliminary test; it is possible that personal skills and knowledge may affect the platform usability and user experience. In addition, the participants only used and assessed the desktop web application, which is why we are planning to evaluate the *SUPER* mobile application to identify the possible differences in usability between the two versions. Moreover, in this work, we mainly followed an unmoderated approach for the usability test: the researchers only introduced the platform, but did not interact with the participants during the test sessions. Therefore, we did not collect completion times for each participant, only establishing a general time frame for the test sessions in order to allow the participants to process the tasks at their own pace. Furthermore, remote testing did not facilitate the collection of these data. Future works will include further collection of quantitative data, such as processing times for each task. Finally, the present study aimed to illustrate the *SUPER* project and to investigate the platform’s preliminary usability, not the clinical efficacy of this approach. Currently, *SUPER* is used in clinical practice by rehabilitation centers that joined the project, but we intend to conduct further studies to evaluate the usefulness and efficacy of the platform, including randomized clinical trials and follow-up studies.

## 5. Conclusions

In the current paper, we introduced a new Italian technological system: *SUPER*, a digital platform that can be used to share uploaded materials and contents on ASD, creating a personalized virtual area of collaboration. This area was designed to support the daily activities of the families, health services, and schools taking care of children with ASD. It provided several sharing and collaborative tools that can be customized based on different requirements. A first preliminary user test with 18 therapists and 12 parents of children with ASD was conducted. None of the participants reported issues examining the contents within the platform and using the variety of tools provided. In fact, both the therapists and parents experienced a high level of system usability.

This work contributes to existing knowledge on telemedicine systems by providing a new multidisciplinary approach that can support all of the different kinds of interventions for children with ASD. We integrated the recommendations from international guidelines into the system design process and developed new tools that were useful to carry out an individual-centered approach. However, the generalizability of our results is subject to certain limitations. To complete the usability evaluation of the *SUPER* platform, future works should: (i) involve the teachers and clinicians of children with ASD; (ii) assess the possible differences between the desktop web application and the mobile application; (iii) investigate the learnability component and how it can influence usability; and (iv) explore whether the users’ personal skills can affect the usability. In addition, we should research the clinical efficacy of our system with a randomized clinical trial. If future investigations are positive, we intend to extend and adapt *SUPER* for teenagers and adults with ASD.

## Figures and Tables

**Table 1:** Different user profiles created in the *SUPER* platform. The table reports the user profiles that could be created depending on the role.

User Profile	User Type
Clinical profile	Pediatrics, neuropsychiatrists, therapists, psychologists, and other clinical professionals
Social and educational profile	Teachers, educators, special needs teachers, social workers, and other social and educational professionals
Family profile	Parents, relatives, caregivers, and legal guardians

**Table 2:** An overview of the different access levels of the Personalized Area based on the profiles. The table shows which sections were available based on the user profile.

Sections of the Personalized Area	User Access
General section	Accessible to all users
Clinical section	Access restricted to users with a clinical profile
Socio-educational section	Access restricted to users with socio-educational and clinical profiles
Family section	Access restricted to users with family and clinical profiles

**Table 3:** Task checklist for the usability test. The table reports the tasks that the users had to perform during their usability test session.

	Tasks
1	Explore the platform
2	Play videos in the General Area
3	Download materials from the General Area
4	Use tools in the Personalized Area

**Table 4:** System usability scale (SUS), English and Italian versions. The table reports the SUS items for English and Italian.

SUS English Version	SUS Italian Version
1. I think I would like to use this system frequently.	1. Penso che mi piacerebbe utilizzare questo sistema frequentemente.
2. I found the system unnecessarily complex.	2. Ho trovato il sistema complesso senza che ce ne fosse bisogno.
3. I thought the system was easy to use.	3. Ho trovato il sistema molto semplice da usare.
4. I think I would need the support of a technical person to be able to use this system.	4. Penso che avrei bisogno del supporto di una persona già in grado di utilizzare il sistema.
5. I found the various functions in this system were well integrated.	5. Ho trovato le varie funzionalità del sistema bene integrate.
6. I thought there was too much inconsistency in this system.	6. Ho trovato incoerenze tra le varie funzionalità del sistema.
7. I would imagine that most people would learn to use this system very quickly.	7. Penso che la maggior parte delle persone potrebbero imparare ad utilizzare il sistema facilmente.
8. I found the system very cumbersome to use.	8. Ho trovato il sistema molto macchinoso da utilizzare.
9. I felt very confident using the system.	9. Ho avuto molta confidenza con il sistema durante l’uso.
10. I needed to learn a lot of things before I could get going with this system.	10. Ho avuto bisogno di imparare molti processi prima di riuscire ad utilizzare al meglio il sistema.

**Table 5:** Summary of SUS scores. The table reports the mean and standard deviations of SUS, usability, and learnability scores, both general and specific, for the two user groups.

Scale	Total Participants	Therapist Group	Parent Group
	(*n* = 30)	(*n* = 18)	(*n* = 12)
SUS	89.2 (SD = 5.5)	90.7 (SD = 5.1)	86.9 (SD = 5.5)
Usability	96.1 (SD = 4.6)	97.1 (SD = 5.0)	94.6 (SD = 3.8)
Learnability	61.7 (SD = 12.5)	65.3 (SD = 9.1)	56.2 (SD = 15.5)

## Abbreviations

The following abbreviations are used in this manuscript:

ASD	Autism spectrum disorder
ICF-CY	International Classification of Functioning, Disability and Health for children and youth
SUPER	Sistema Unitario in una Piattaforma Riabilitativa ed Educativa
SUS	System usability scale

## References

[1] American Psychiatric Association (2013). Diagnostic and Statistical Manual of Mental Disorders (DSM-5®).

[2] Chita-Tegmark M. (2016). Social attention in ASD: A review and meta-analysis of eye-tracking studies. Res. Dev. Disabil..

[3] Mouga S., Castelhano J., Café C., Sousa D., Duque F., Oliveira G., Castelo-Branco M. (2021). Social Attention Deficits in Children with Autism Spectrum Disorder: Task Dependence of Objects vs. Faces Observation Bias. Front. Psychiatry.

[4] Purpura G., Tagliabue L., Petri S., Cerroni F., Mazzarini A., Nacinovich R. (2021). Caregivers’ Burden of School-Aged Children with Neurodevelopmental Disorders: Implications for Family-Centred Care. Brain Sci..

[5] Davis N.O., Carter A.S. (2008). Parenting stress in mothers and fathers of toddlers with autism spectrum disorders: Associations with child characteristics. J. Autism Dev. Disord..

[6] Baykal S., Karakurt M.N., Çakır M., Karabekiroğlu K. (2019). An examination of the relations between symptom distributions in children diagnosed with autism and caregiver burden, anxiety and depression levels. Community Ment. Health J..

[7] Diggle T., McConachie H. (2002). Parent-mediated early intervention for young children with autism spectrum disorder. Cochrane Database Syst. Rev..

[8] Hyman S.L., Levy S.E., Myers S.M., Council on Children with Disabilities, Section on Developmental and Behavioral Pediatrics (2020). Identification, Evaluation, and Management of Children with Autism Spectrum Disorder. Pediatrics.

[9] Ellerbeck K., Smith C., Courtemanche A. (2015). Care of Children with Autism Spectrum Disorder. Prim. Care Clin. Off. Pract..

[10] Scottish Intercollegiate Guidelines Network (SIGN) (2016). Assessment, Diagnosis and Interventions for Autism Spectrum Disorders.

[11] Fuentes J., Hervás A., Howlin P. (2021). ESCAP practice guidance for autism: A summary of evidence-based recommendations for diagnosis and treatment. Eur. Child Adolesc. Psychiatry.

[12] National Institute for Health and Care Excellence (UK) (2013). Autism Spectrum Disorder in under 19s: Support and Management (NICE Clinical Guideline 170). https://www.nice.org.uk/guidance/cg170.

[13] Whitehouse A., Evans K., Eapen V., Prior M., Wray J. (2017). The Diagnostic Process for Children, Adolescents and Adults Referred for Assessment of Autism Spectrum Disorder in Australia: A National Guideline (Draft Version for Community Consultation). https://www.autismcrc.com.au/sites/default/files/inline-files/Australian%20National%20Guideline%20for%20ASD%20Assessment%20-%20Draft%20Version%20for%20Community%20Consultation_0.pdf.

[14] Myers S.M., Johnson C.P. (2007). Management of Children with Autism Spectrum Disorders. Pediatrics.

[15] Palisano R.J., Di Rezze B., Stewart D., Rosenbaum P.L., Hlyva O., Freeman M., Nguyen T., Gorter J.W. (2017). Life course health development of individuals with neurodevelopmental conditions. Dev. Med. Child Neurol..

[16] Hayes M.J. (2015). An evaluation of the impacts of the Social Communication Emotional Regulation and Transactional Support (SCERTS^®^) Model on multidisciplinary collaboration. Int. J. Cross-Discip. Subj. Educ..

[17] World Health Organization (2020). Statement on the Second Meeting of the International Health Regulations. Emergency Committee Regarding the Outbreak of Novel Coronavirus (2019-nCoV). https://www.who.int/news/item/30-01-2020-statement-on-the-second-meeting-of-the-international-health-regulations-(2005)-emergency-committee-regarding-the-outbreak-of-novel-coronavirus-(2019-ncov).

[18] Narzisi A. (2020). Phase 2 and Later of COVID-19 Lockdown: Is it Possible to Perform Remote Diagnosis and Intervention for Autism Spectrum Disorder? An Online-Mediated Approach. J. Clin. Med..

[19] Conti E., Sgandurra G., De Nicola G., Biagioni T., Boldrini S., Bonaventura E., Buchignani B., Della Vecchia S., Falcone F., Fedi C. (2020). Behavioural and Emotional Changes during COVID-19 Lockdown in an Italian Paediatric Population with Neurologic and Psychiatric Disorders. Brain Sci..

[20] Dhiman S., Sahu P.K., Reed W.R., Ganesh G.S., Goyal R.K., Jain S. (2020). Impact of COVID-19 outbreak on mental health and perceived strain among caregivers tending children with special needs. Res. Dev. Disabil..

[21] Conti E., Chericoni N., Costanzo V., Lasala R., Mancini A., Prosperi M., Tancredi R., Muratori F., Calderoni S., Apicella F. (2020). Moving Toward Telehealth Surveillance Services for Toddlers at Risk for Autism During the COVID-19 Pandemic. Front. Psychiatry.

[22] Daulay N. (2021). Home education for children with autism spectrum disorder during the COVID-19 pandemic: Indonesian mothers experience. Res. Dev. Disabil..

[23] Desideri L., Pérez-Fuster P., Herrera G. (2021). Information and Communication Technologies to Support Early Screening of Autism Spectrum Disorder: A Systematic Review. Children.

[24] Gevarter C., Najar A.M., Flake J., Tapia-Alvidrez F., Lucero A. (2021). Naturalistic Communication Training for Early Intervention Providers and Latinx Parents of Children with Signs of Autism. J. Dev. Phys. Disabil..

[25] Wagner L., Corona L.L., Weitlauf A.S., Marsh K.L., Berman A.F., Broderick N.A., Francis S., Hine J., Nicholson A., Stone C. (2020). Use of the TELE-ASD-PEDS for Autism Evaluations in Response to COVID-19: Preliminary Outcomes and Clinician Acceptability. J. Autism Dev. Disord..

[26] Pecini C., Spoglianti S., Michetti S., Bonetti S., Di Lieto M.C., Gasperini F., Cristofani P., Bozza M., Brizzolara D., Casalini C. (2018). Telerehabilitation in developmental dyslexia: Methods of implementation and expected results. Minerva Pediatr..

[27] Provenzi L., Grumi S., Borgatti R. (2020). Alone with the kids: Tele-medicine for children with special healthcare needs during COVID-19 emergency. Front. Psychol..

[28] Faccioli S., Lombardi F., Bellini P., Costi S., Sassi S., Pesci M.C. (2021). How did Italian adolescents with disability and parents deal with the COVID-19 emergency?. Int. J. Environ. Res. Public Health.

[29] World Health Organization (2007). International Classification of Functioning, Disability and Health: Children & Youth Version: ICF-CY.

[30] Brooke J. (1996). SUS: A quick and dirty usability scale. Usabil. Eval. Ind..

[31] Aldred C., Taylor C., Wan M., Green J., Siller M., Morgan L. (2018). Using video feedback strategies in parent-mediated early autism intervention. Handbook of Parent-Implemented Interventions for Very Young Children with Autism.

[32] Modular Object-Oriented Dynamic Learning Environment—MOODLE. https://moodle.org.

[33] Gogan M.L., Sirbu R., Draghici A. (2015). Aspects Concerning the Use of the Moodle Platform—Case Study. Procedia Technol..

[34] The European Parliament (2016). Regulation (EU) 2016/679 of the European Parliament and of the Council of 27 April 2016 on the Protection of Natural Persons with Regard to the Processing of Personal Data and on the Free Movement of Such Data, and Repealing Directive 95/46/EC (General Data Protection Regulation). https://eur-lex.europa.eu/eli/reg/2016/679/oj.

[35] Borsci S., Federici S., Lauriola M. (2009). On the dimensionality of the System Usability Scale: A test of alternative measurement models. Cogn. Process..

[36] Bangor A., Kortum P., Miller J. (2009). Determining what individual SUS scores mean: Adding an adjective rating scale. J. Usabil. Stud..

[37] Othman M.K., Nogoibaeva A., Leong L.S., Barawi M.H. (2021). Usability evaluation of a virtual reality smartphone app for a living museum. Univers. Access Inf. Soc..

[38] Lewis J.R., Sauro J. (2009). The factor structure of the system usability scale. Proceedings of the International Conference on Human Centered Design.

[39] Golomb M.R., Mcdonald B.C., Warden S.J., Yonkman J., Saykin A.J., Shirley B., Huber M., Rabin B., Abdelbaky M., Nwosu M.E. (2010). In-Home Virtual Reality Videogame Telerehabilitation in Adolescents with Hemiplegic Cerebral Palsy. Arch. Phys. Med. Rehabil..

[40] Tinelli F., Cioni G., Purpura G. (2017). Development and Implementation of a New Telerehabilitation System for Audiovisual Stimulation Training in Hemianopia. Front. Neurol..

[41] Philpott-Robinson K., Lane A.E., Harpster K. (2016). Sensory features of toddlers at risk for autism spectrum disorder. Am. J. Occup. Ther..

[42] Purpura G., Costanzo V., Chericoni N., Puopolo M., Scattoni M.L., Muratori F., Apicella F. (2017). Bilateral Patterns of Repetitive Movements in 6- to 12-Month-Old Infants with Autism Spectrum Disorders. Front. Psychol..

[43] LeBarton E.S., Landa R.J. (2019). Infant motor skill predicts later expressive language and autism spectrum disorder diagnosis. Infant Behav. Dev..

[44] Apicella F., Costanzo V., Purpura G. (2020). Are early visual behavior impairments involved in the onset of autism spectrum disorders? Insights for early diagnosis and intervention. Eur. J. Pediatr..

